# A Novel Location-Centric IoT-Cloud Based On-Street Car Parking Violation Management System in Smart Cities

**DOI:** 10.3390/s16060810

**Published:** 2016-06-02

**Authors:** Thanh Dinh, Younghan Kim

**Affiliations:** 1 School of Electronic Engineering, Soongsil University, Sangdo-dong, Dongjak-Gu, Seoul 06978, Korea; thanhdcn@dcn.ssu.ac.kr; 2 Room 1104, Huyngam Engineering Building 424, Soongsil University, Sangdo-do, Dongjak-Gu, Seoul 06978, Korea

**Keywords:** Internet of Things, IoT cloud, sensor network, parking management system, parking violation management system, smart cities

## Abstract

Nowadays, in big cities, parking management is a critical issue from both the driver’s side and the city government’s side. From the driver’s side, how to find an available parking lot in a city is a considerable concern. As a result, smart parking systems recently have received great interest, both in academia and industry. From the city government’s side, how to manage and distribute such a limited public parking resource efficiently to give every visitor a fair chance of finding an on-street parking lot is also a considerable concern. However, existing studies of smart parking management focus only on assisting the driver’s side to find available parking spaces. This study aims to raise a new perspective on such smart parking management and to propose a novel location-centric IoT-cloud-based parking violation management system. The system is designed to assist authoritative officers in finding parking violations easily and recommends the least cost path for officers so that officers can achieve their highest productivity in finding parking violations and issuing parking tickets. Experimental results show that the system not only improves the productivity of officers in finding parking violations and issuing tickets, but also helps reduce the traveling cost of officers and to reduce the average violation period of violating cars considerably.

## 1. Introduction

Nowadays, applications of the Internet of Things (IoT) to urban environments are receiving great interest because they help push city governments to utilize ICT solutions in managing public affairs, thus enabling the so-called smart city [1]. Although the concept of the smart city is not yet popular in real life, the main purpose of such a research trend is to make efficient use of public resources, improving the quality of services provided to city citizens and minimizing the operating costs of public administrations.

In big cities, parking management is a critical issue for both driver’s side and city government’s side. From the driver’s side, how to find an available parking lot in a city is a considerable concern. As a result, in the trend of smart cities [1], smart parking systems [2,3,4,5] recently have received great interest. Current studies on this topic focus on supporting drivers to easily find an available parking space [6,7]. In smart parking systems, the Internet of Things (*i.e*., wireless sensor networks) is normally used to provide parking sensing service for managing parking lots, detecting available parking lots and increasing the probability of finding vacant parking spaces or even reserving a parking lot [6,8,9,10]. From the city government’s side, how to manage and distribute a limited public parking resource efficiently to give every visitor a fair chance of finding an on-street parking lot is also a considerable concern. For government officers, parking violation management is a challenge. The concerns from both sides are common and worth investigating. However, existing studies on this topic focus only on smart parking management to assist the driver’s side. In this paper, we are interested in exploring the parking sensing service to assist the city government’s side, instead of the driver’s side. In particular, we investigate a new perspective of smart parking management to support government officers on managing on-street parking violations.

On-street parking spaces are very important to cities [11], especially big cities with a high number of visitors daily, like Seoul, New York, Melbourne, Singapore, *etc*. In such big cities, demand for on-street parking is extremely high [12]. With a limited number of on-street parking lots inside a city, city authorities have to manage those resources well to address the demands of most visitors. Parking rules (*i.e*., parking time restrictions and enforcement) are thus enacted. For example, on a street A, from 8 a.m. to 8 p.m., the maximum period a car can park is 2 h. Parking rules are very important to city authorities to make parking spaces regularly turnover and to ensure on-street parking systems work fairly for every visitor. Without those restrictions or inefficient enforcing of parking rules, on-street parking spaces may be occupied by the same vehicles for a long period of time (*i.e*., parking violating cars), thus creating significant inconvenience and reducing the parking availability to others. This kind of unfair resource distribution may lead to other issues, such as traffic congestion, difficulties for visitors to find a parking lot to go shopping or dining, wasting the time and money of visitors, *etc*. As a result, visitors may feel unhappy and unsatisfied when visiting a city. For that reason, many visitors may not come back to the city again for shopping, dining or other businesses.

Due to the considerably negative impact of parking violations, big cities normally have a heavy fine policy for parking violations. Along with a heavy fine policy, government officers are required to manage parking violations well. However, in many cities, parking officers currently find violating cars through luck and experience, so the number of parking violations that is not handled properly still remains great [13]. With an inefficient method, a limited number of parking officers cannot take control of parking violations efficiently. This causes the number of parking violations to remain high in many cities. For that concern, it is crucial to look for an efficient solution to enable parking officers to easily search for violating cars and to process them efficiently. An efficient method to handle parking violations is very important to maintain the attraction of a city for visitors and shoppers, affecting the economic development of the city. In addition, with an efficient method, parking officers can also help increase their city budget from parking fines, which normally have a significant ratio in the total budget of big cities.

This paper proposes a novel location-centric IoT-cloud-based parking violation management system to assist parking officers in managing and handling parking violations efficiently, which is one of the first attempts in this direction to enable the so-called smart city. To provide actual views about parking violations in big cities, we first carry out an extensive analysis for parking violations based on a massive dataset of real parking events obtained by parking sensors in the City of Melbourne [14]. The dataset was recently published by the Victoria State government. Sensor data records are gathered from in-ground parking sensors deployed around the city [14,15,16], which are currently used for parking fee management purposes. Instead of analyzing the sensor dataset for parking fee management purposes, we are interested in extracting parking violation information from the sensor dataset. Our sensor data analysis provides a comprehensive picture, as well as the temporal and spatial distribution of parking violations in a city. This information later enables us to design an efficient architecture and model for the PVMsystem.

Based on the understanding of the parking violation distribution, we propose an architectural model and design an efficient PVM system. In the system, parking violations are detected through sensor records forwarded to a sensor cloud. Parking violation information is then distributed to officers based on a location-centric approach to help them find parking violations easily. Based on available parking violation information and the current location of an officer, the system computes and recommends the least cost path to enable the officer to efficiently handle parking violations. In theoretical aspects, we model the system as a network graph and the officer path finding problem as the least cost forwarding problem. We then solve the problem by proposing a least cost path recommendation algorithm. Results from our extensive simulations using real sensor dataset show that our PVM system helps (1) improve the productivity of officers in finding parking violations and issuing tickets, (2) reduce their traveling cost, as well as (3) reduce the average violation period of violating cars considerably.

In summary, this paper makes the following contributions.

- We raise a new perspective of smart parking management for the city government’s side and highlight the necessity of an efficient parking violation management system to assist government officers in managing parking violations.
- We analyze a massive dataset of real parking events obtained from in-ground parking sensors to provide actual views and spatial/temporal distributions of parking violations in big cities.
- We design a novel location-centric IoT-cloud-based parking violation management system with proposed mechanisms to assist officers in managing parking violations efficiently.
- We conduct extensive experiments based on a real parking sensor dataset. The obtained results show that the system not only improves the productivity of officers in finding parking violations and issuing tickets, but also reduces the traveling cost of officers and reduces the average violation period of violating cars considerably.

The rest of this paper is organized as follows. Section 1 discusses related works. Section 2 presents the parking sensor data analysis results. Section 3 describes our proposed system. Section 4 gives details about the system implementation and shows evaluation results. Finally, in Section 5, we discuss the limitations of the current work, possible extensions in this topic for future works and conclude the paper.

## 2. Related Work

Among applications for a smart city [1,17,18,19], a smart parking system [20,21,22] has received great interest from both academia and industry. Barone *et al*. [23] proposes an intelligent parking assistant architecture for efficient public parking management systems. Based on the architectural design, drivers can obtain information about on-street parking availability and reserve parking spaces. In [24], Guo and Li find a relationship between a vehicular-communication traffic control system and a smart parking system. Based on the findings, the authors exploit traffic data in cyber physical systems and intelligent transportation systems to improve the efficiency of smart parking systems. A multivariate spatial-temporal prediction model [25] is proposed to further increase the probability that a driver can find available parking spaces.

In most of the smart parking management studies, the parking sensor network [26] is an important component [3,10,16]. Those smart parking management systems utilize sensor networks in the bottom layer to manage the occupation status of parking lots for both on-street and off-street parking. In the parking sensor network, sensors are deployed in the ground of parking lots to detect whether parking lots are available or occupied by vehicles. The detection of in-ground parking sensors may be based on various techniques [3,8,9,27]. Based on sensing information, smart parking management systems provide sensing services to drivers about the availability of parking spaces. Recently, several big cities have deployed such a sensor network for smart parking management, including Melbourne city [2,13,14].

In traditional approaches based on vehicle-to-vehicle and sensor-to-vehicle [28] parking systems aim to provide information about nearby parking spaces for drivers. Recently, cloud-based smart parking management systems [2,3] have become a hot trend due to the advantages of the cloud in storing, processing and distributing parking information [21] or even parking reservations [6].

Although current smart parking management system solutions provide considerable benefits to users, literature studies only focus on assisting drivers in finding available parking spaces. In other words, current smart parking management systems provide only solutions for the driver’s/citizen’s side. To the best of our knowledge, there is no available studies that focus on assisting city government officers to manage public parking spaces (*i.e*., on-street parking violations). In public parking management, parking violation management is a critical issue, which is nowadays a great concern of many city governments because they are required to distribute a limited on-street parking resource equally to a huge number of visitors each day. Due to the importance of parking violation management, this paper investigates a novel perspective of smart parking management systems to assist government officers to manage parking violations efficiently.

## 3. On-Street Parking Sensor Data Analysis: From Sensing to Parking Violation Information

Most of studies on smart on-street parking management systems share a similar design in wireless sensor network deployment [2,3,9,10]. The parking sensor network deployed in Melbourne is not an exception [14]. In particular, each parking bay is deployed with an in-ground parking sensor. Each parking area consists of a number of parking bays with a master node, which gathers sensing data from in-ground parking sensors in the parking area. In this section, we analyze a massive parking sensor dataset of Melbourne, as an example. The purpose is to provide actual views about on-street parking violations. First, we provide an overview about parking rules and parking fines of the city.

### 3.1. On-Street Parking Rules and Parking Fines

The city government has built thousands of on-street parking bays around the city. However, with about nearly one million visitors a day, how to distribute a limited number of on-street parking spaces fairly for all visitors and to control traffic problems is a critical issue of the city government. A set of strict rules and a heavy fine policy have been established for that purpose. Parking rules define the maximum allowable parking period in different time frames in a day for parking areas.

However, the parking violation analysis results in Section 3.2 indicate that parking rules are not followed well, and the number of parking violations in the city still remains great over the years. The reason may be that with a limited number of officers patrolling randomly around the city, only a limited number of violating cars are processed, and a major portion of parking violations is not handled properly. A great number of parking violations may be one of the main reasons that has led to the fact that visitors find it difficult to look for available parking spaces in the city. If parking violations are managed well, this problem can be alleviated.

### 3.2. Sensor Data Analysis for Parking Violation Information

**Figure 1:**
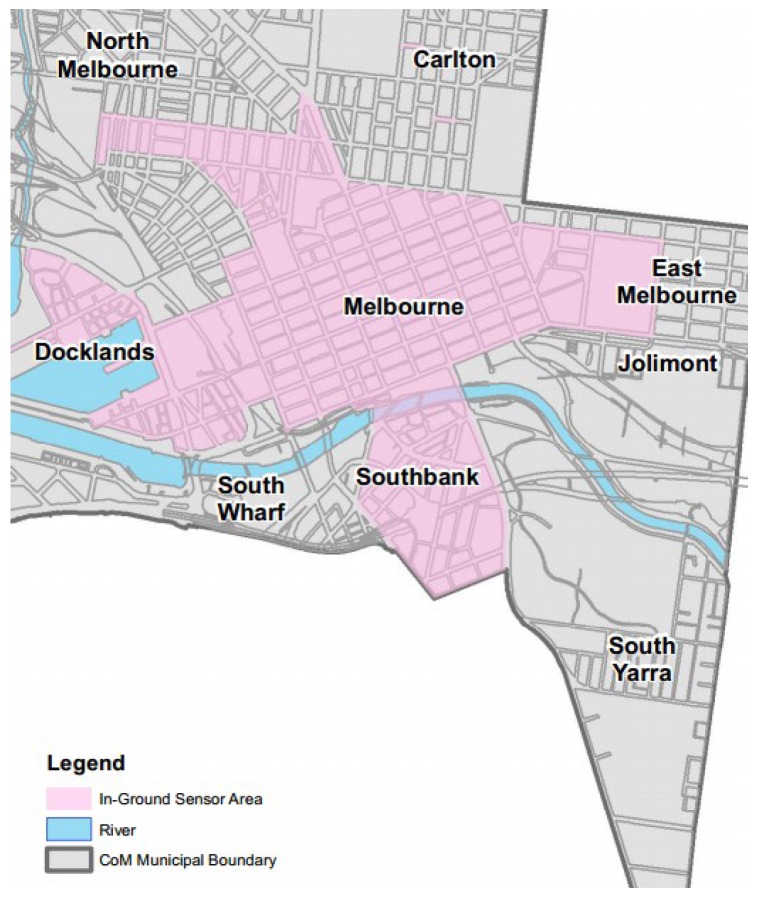
Melbourne in-ground parking sensor map. Source: Victoria State Government, City of Melbourne [14].

For parking fee management, in-ground parking sensors are deployed at more than 4600 parking bays around the city, as shown in Figure 1. Each sensor detects when a parking bay is occupied and its parking duration. Sensing events from sensors are reported to a master node (*i.e*., a master node can be integrated with ticket machines or meters) nearby. Recently, the Victoria State government opened a massive dataset of parking sensor events to the public. The dataset consists of a huge number of sensor events with over 12 million records. Instead of investigating the dataset for parking fees or smart parking management, we are more interested in finding a new perspective from the dataset. In particular, we extract the parking violation distribution from the dataset to provide actual views about parking violations in the city and to design a system to assist officers in finding and catching parking violations.

**Figure 2:**
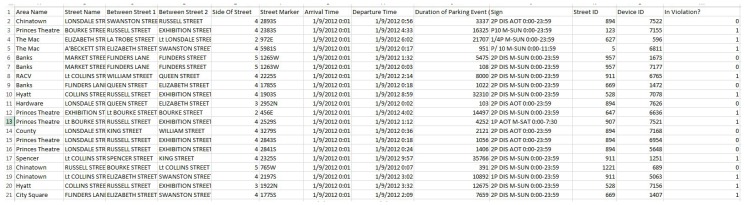
A sample of parking sensor data (source: Victoria State Government, City of Melbourne) and violation detection based on sensor data.

Each sensor record R from a parking sensor consists of the following attributes R (device ID, occupation status (1 or 0), vehicle arrival time, vehicle departure time). We also obtain a location dataset of sensors from the government [14]. In our analysis, the ID of a sensor node is associated with sensor location (longitude, latitude) and the parking area, which is represented by the master node. The location of a sensor is associated with more detailed information, including area name, street name, street segment, street marker and parking sign at that street segment. A parking sign can be matched with a set of corresponding parking rules stored in a database. By comparing the parking information of a car (*i.e*., parking duration) reported by sensors and parking rules at the corresponding parking area, we can detect parking violations. A sample of parking sensor data and violation detection based on sensor data is illustrated in Figure 2.

Parking violation : A car that is parked is called a parking violation when it does not comply with the parking rules. For example, its parking duration is over the permitted period.

We group violation events based on temporal and spatial dimensions to visualize violation distributions. In the spatial dimension, violations are clustered based on street segment and area. In the temporal dimension, violations are grouped based on time of a day and day of a week. The purpose is to recognize violation patterns in spatial and temporal dimensions to assist city authorities in managing parking violations efficiently.

#### 3.2.1. Spatial Distribution Analysis of Parking Violations

**Figure 3:**
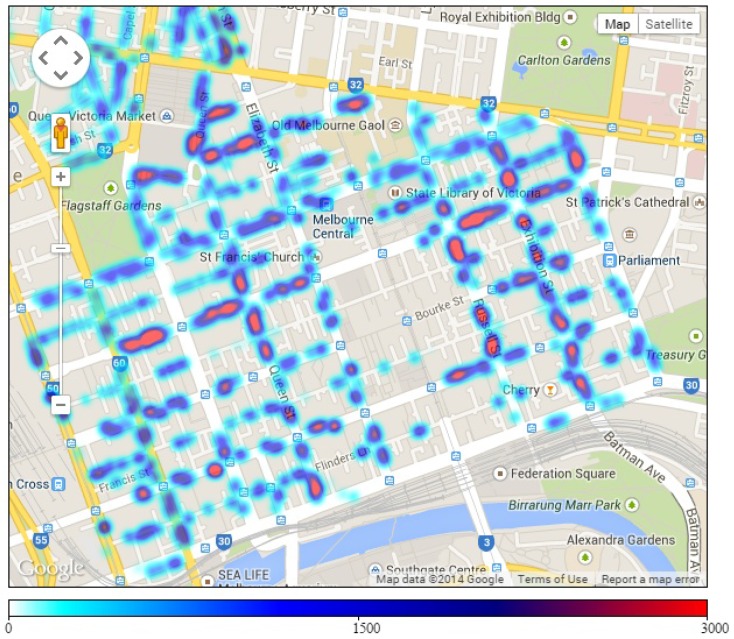
Parking volume map for each street segment.

Figure 3 shows the geographical distribution of yearly parking volume (*i.e*., the number of cars parked in a position) in different streets around the city. The obtained results are drawn on the Google map of the city. Street segments with on-street parking bays are highlighted with colors. Some street segment may not have on-street parking spaces. The darkness level of colors indicates a low or high volume of parking. A lighter color (*i.e*., bright blue) implies less parking and a darker color (*i.e*., dark red) implies more parking. The number of parking recorded in some positions is over 3000. We can see that a high car parking volume concentrates in areas near St. Francis Church on Lonsdale street and areas around the State Library of Victoria on Russel street. The number of parked cars in different locations has a great variation.

**Figure 4:**
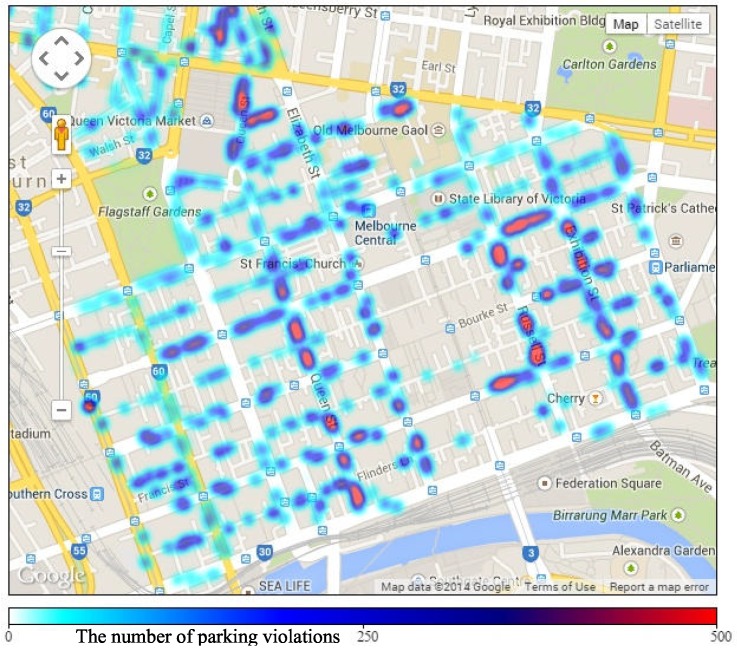
Parking violation volume map for each street segment.

Figure 4 visualizes the geographical distribution of the number of parking violations, in comparison with the parking volume shown in Figure 3. The same colors are used to present levels of violation volume (light colors for low volume and dark colors for high volume). Overall, the distribution of the parking violation volume has a strong coherence with the distribution of the parking volume. However, some areas with a high volume of parked cars do not necessarily have a high volume of parking violations. For example, although the street segment on Lonsdale street near St. Francis Church and that on Collins street from Queen to Francis street have a great number of parked cars, a low number of parking violations are witnessed. The figure shows that for some positions, the number of parking violations is over 500, while some positions witness less than 50 violations. The violation densities of different areas are various. The parking violation distribution presented in this figure can help the city government in planning and distributing human resources efficiently in different areas for parking violation management (*i.e*., distributing the number of parking officers in proportion with the parking violation volume).

**Figure 5:**
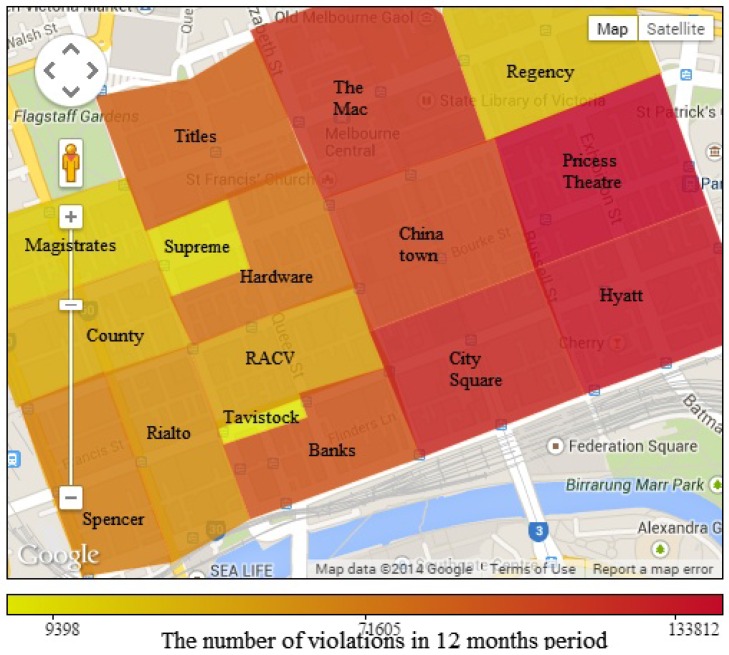
Parking violation distribution by areas.

**Figure 6:**
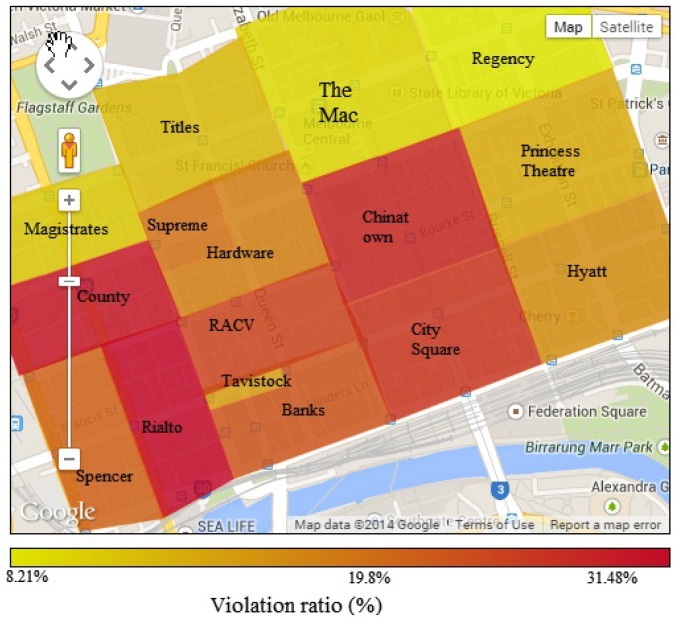
Parking violation ratio by areas.

We group violation data on different areas, which are based on political areas and similarity in parking violation distribution. Results are presented in Figure 5 and Figure 6. Figure 5 maps the number of parking violations in different areas. The highest number of parking violations happens around Princess, Hyatt and City Square areas with more than 130,000 violations yearly in each area. The number of parking violations in the city is really huge, while the number of parking violating cars penalized each year is very small compared to the total number. This highlights a need to improve the city’s parking violation management method. The Mac and Chinatown also witness a high number of violations, while areas on the left have a lower number of parking violations. The violation distribution in each area is clearly distinguished. According to the parking violation distribution, the city government can allocate human resources efficiently for each area.

Figure 6 visualizes the parking violation ratio (*i.e*., the ratio between the number of parking violations and the parking volume) of the areas, as presented in Figure 5. By comparing Figure 5 and Figure 6, we find that an area with a high number of violations does not necessarily have a high parking violation ratio. While areas on the left have a low number of parking violations, their parking violation ratios are very high, up to 31.48 %. On the other hand, areas with a high number of parking violations like Princess Theatre and Hyatt surprisingly have a low parking violation ratio. The sensor data analysis results help the city authorities to set targets to reduce the total number of parking violations or to reduce the parking violation ratio in each area.

#### 3.2.2. Temporal Distribution Analysis of Parking Violations

We now analyze the temporal distribution of parking violations based on the parking sensor data.

**Figure 7:**
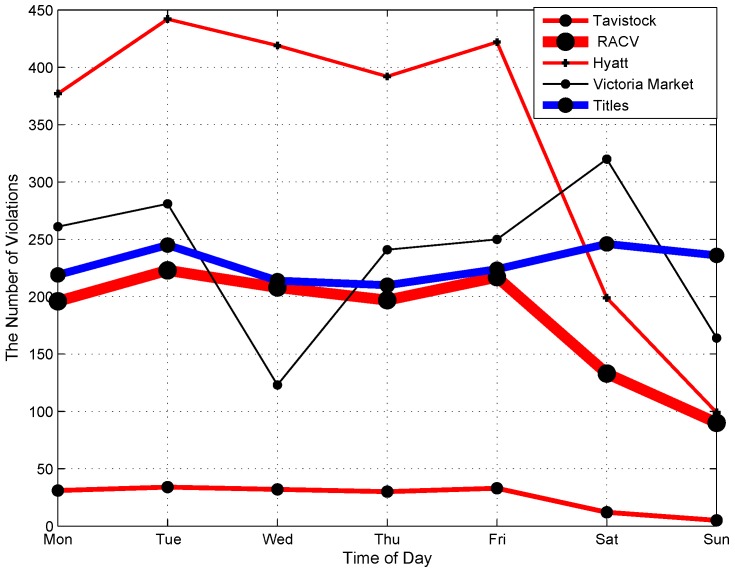
Temporal distribution of parking violations on different days in a week.

Figure 7 presents the average number of parking violations in main areas on different days in a week. We find five main trends in the number of parking violations on days in a week. Graphs of the five areas, including Tavistock, RACV, Hyatt, Queen Victoria Market and Titles, are representative of the five trends. In particular, the graph of Hyatt area fluctuates and remains at a high level during weekdays and goes down to a low level during weekends. In the RACV area, the number of parking violations remains stable at a medium level during weekdays and declines on weekends. A similar characteristic is found on the graph of Tavistock, but the number of violations in this area remains at a low level during the week. In the Titles area, the number of parking violations increases significantly on weekends compared to weekdays. In Queen Victoria Market area, the number of parking violations decreases on Wednesday and Sunday when the market closes. Note that Queen Victoria Market is located near the Titles area as shown in Figure 3 and Figure 4. There are different trends in different areas possibly due to the functioning and characteristics of each area. Some areas mainly serve offices and companies, which normally do not open on weekends, while other areas may serve as shopping centers, which are busier on weekends.

**Figure 8:**
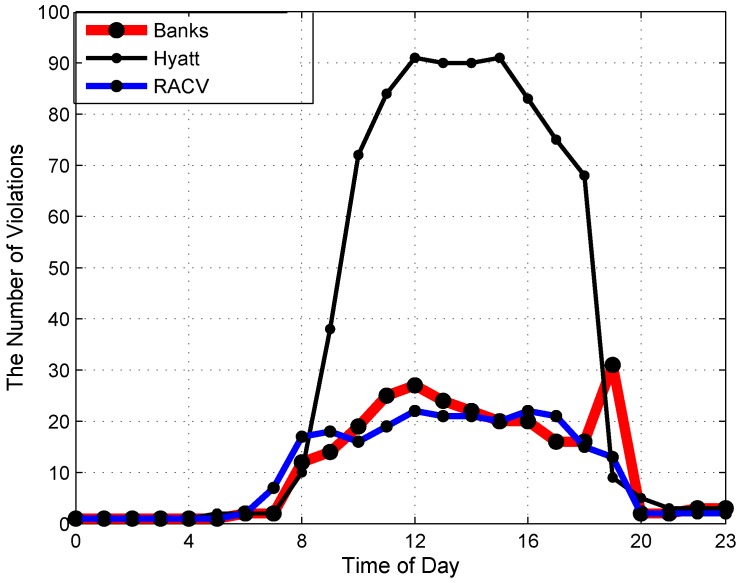
Temporal distribution of parking violations at different times in a day.

Figure 8 shows the main trends of average parking violations at different times in a day with three representative areas. In most areas, the number of parking violations increases from 8 a.m., achieves a high volume around 12 p.m. and decreases to a low volume from 8 p.m. However, in shopping areas like Banks, the highest number of parking violations happens around 6 p.m. to 8 p.m., instead of 12 p.m., like other areas.

The temporal distribution of parking violations in each area as presented in the Figure 7 and Figure 8 is meaningful to enable the city government to schedule working shifts of officers efficiently in proportion with the volume of violations at different times of a day and different days of a week.

**Figure 9:**
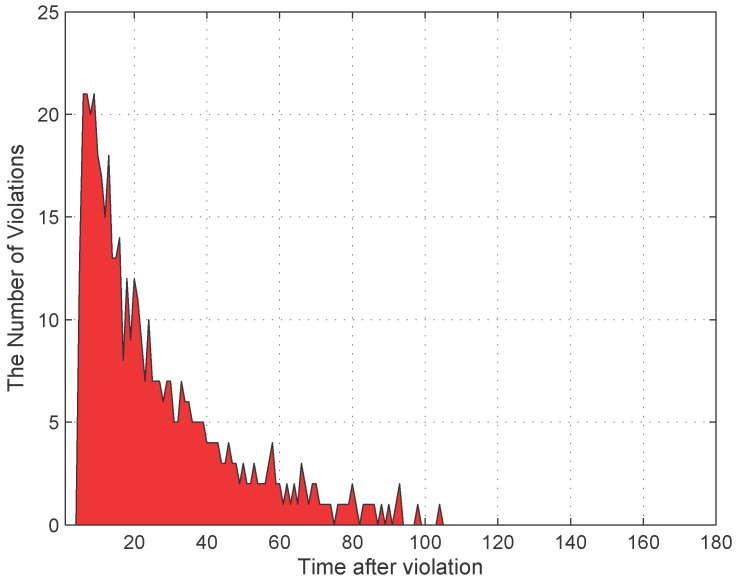
The distribution of average violation periods.

Figure 9 presents the distribution of average violation periods, which are measured from the time a car starts violating until the time the car leaves or is penalized. Most of the violating cars violate with a period from 5 min to 60 min. From sensor data, we can calculate such a distribution for different areas of the city. The results for the distribution of average violation periods in an area enable an officer to estimate the probability a violating car may leave before the officer visits its parking location.

## 4. Location-Centric IoT-Cloud-Based Parking Violation Management System

**Figure 10:**
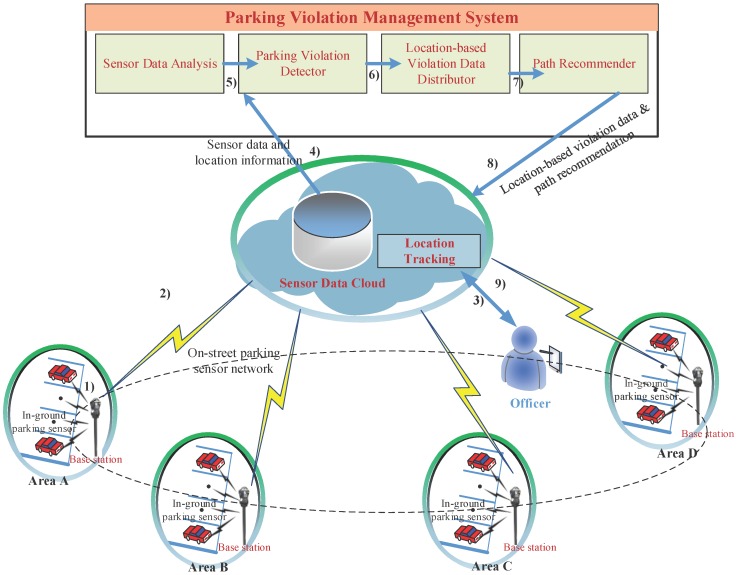
Architectural model of the proposed parking violation management system.

According to the sensor data analysis, the distribution of parking violations highly depends on location, and the distribution in an area is also dynamically changed over time. As a result, for efficient parking violation management, officers should be aware of the parking violation distribution in each area in real time. In addition, any path recommendation for an officer to find and handle parking violations should be made based on the current location of the officer. For this reason, we propose a novel location-centric IoT-Cloud-based parking violation management system. We first define the main entities used in the system model, as shown in Figure 10.

### 4.1. Entities

**Physical parking sensor network**: A physical parking sensor network consists of multiple sub-networks. Physical sensor nodes and a master node in a parking area form a sub-network. Each sensor *i* is characterized by the following properties: ID, location (longitude, latitude), state (1, 0) where 1 is active and 0 is inactive. Each sensor data message will include the node ID, occupation state *δ* (δ=1: occupied, δ=0 : free), vehicle arrival time $T_{a}$ and vehicle departure time $T_{d}$. From the arrival time and the vehicle departure time, we can calculate parking duration $T_{parking}$ as follows.

(1) $$T_{parking}=\left\{\begin{array}{cc} T_{current}-T_{a} & \;\text{if}\;T_{d}=0\\ T_{d}-T_{a} & \;\text{otherwise} \end{array}\right.$$

**Cloud C**: a cloud c is characterized by the following properties: ID, resources and QoS metrics. For simplicity, this paper considers a single cloud and does not consider a selective model for clouds. Therefore, we do not cover the properties of a cloud in detail. We define some basic properties to reserve extensions of this model for a multi-cloud in the future. For management purposes of the city government, private clouds are normally used. A cloud is used as a sink node, which gathers all sensor data from parking sensors, namely the sensor cloud or IoT-cloud. The sensor cloud stores parking information, tracking the locations of users, and provides computing resources for the system.

**Mobile user (*****i.e*****., officer)**, who plays a role as the client side of the system and is characterized by ID (*i.e*., device ID or officer ID), traveling mode and current location. In a more complex model, moving speed and other factors can be considered.

**Application**: A parking violation management application is deployed on the top of a sensor cloud and exploits cloud resources for computing. The application receives sensor data from the sensor cloud and performs analysis based on sensor data. A module, namely the parking violation detector, detects parking violations from the results of sensor data analysis. The parking violation is then grouped by a distributor based on location information and distributed to the client side-based (*i.e*., officers) current location of officers. Finally, a path recommender runs algorithms to determine the best path for officers to find parking violations with a high probability and potentially high number of parking violations.

### 4.2. Processing Flow of the System: From Sensing Data to Meaningful Information

**Figure 11:**
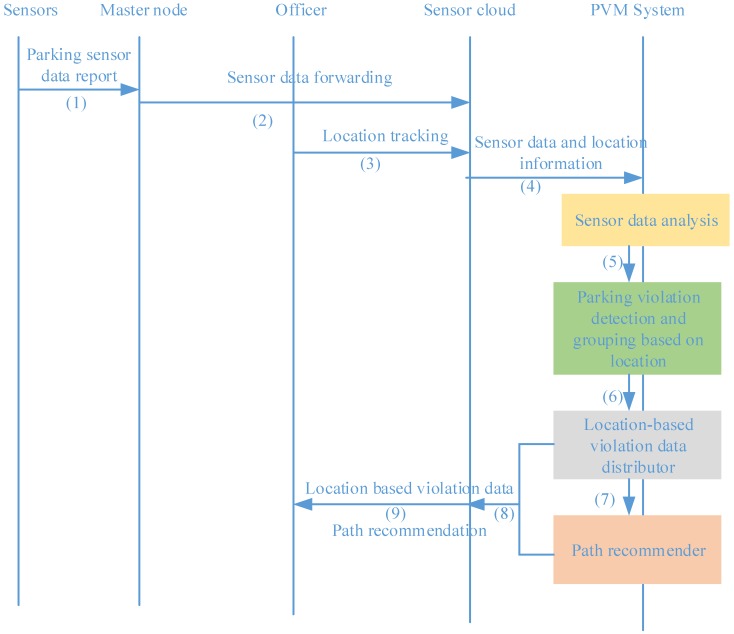
Processing flow of the system.

The processing flow of the system is presented in Figure 11. The inputs of the system are parking sensor data, which are gathered from sensors and forwarded to the cloud by master nodes, and the location of officers obtained through location tracking. After processing on a cloud by the violation detector, the distributor and the recommender as described in the previous section, the outputs of the system are parking violation distribution based on locations and recommendations of the best path for officers to handle violations.

### 4.3. System Parameters

- NV(x): the number of parking violations at cluster node *x*
- $NB_{p}$: the number of parking bays at parking area *P*
- *P*: parking area
- *C*: cluster node
- *b*: parking bay

### 4.4. System Modeling

We assume that based on the distribution of parking violations presented above, the city government divides the city into *R* regions. Each region is managed by an officer. In real working environments, officers also normally work following the task and region division of the city government. An automatic planning tool for city governments to do such a task and region division is out of the scope of this paper and reserved for future works. This paper focuses on designing a system to enable each officer *O* to manage parking violations in his/her area efficiently.

We model the parking violation management system on cloud as a network [3], which is presented in detail as follows. We assume the system consists of *N* on-street parking areas. Each parking area $P_{i}$ consists of $NB_{P_{i}}$ parking bays with in-ground parking sensors belonging to the same master node.

(2) $$P_{i}=\left\{b_{1},b_{2},\ldots ,b_{NB_{P_{i}}}\right\}$$

For the system modeling, we virtualize each on-street parking area $P_{i}$ as a cluster node $C_{i}$ of a network. As a result, we transform the system into a network of *N* cluster nodes. We aggregate the number of parking violations of all parking bays $b_{1},b_{2},\ldots ,b_{NB_{P_{i}}}$ in a parking area $P_{i}$ into the number of parking violations at the node $C_{i}$ as follows.

(3) $$NV\left(C_{i}\right)=NV\left(P_{i}\right)=\sum_{j=1}^{NB_{P_{i}}}NV(b_{j})$$

The reason for the aggregation operation is that parking bays belonging to the same car park are very close to each other. For efficient parking violation handling, once an officer visits a parking area, he or she should handle all current violations in the parking area. In this way, within a moving cost from a parking area to another parking area, an officer can handle multiple violations. As the distance between parking bays in the same parking area is very small compared to the distance between parking areas, we assume the moving cost between parking bays in the same parking area is approximately equal to zero. As a result, we have:

- Cluster node $C_{1}$ corresponding with parking area $P_{1}$ has $NV(C_{1})$ parking violations at the current time.
- Cluster node $C_{2}$ corresponding with parking area $P_{2}$ has $NV(C_{2})$ parking violations at the current time.
- Cluster node $C_{i}$ corresponding with parking area $P_{i}$ has $NV(C_{i})$ parking violations at the current time.

The total number of parking violations NV in the system at time *t* is calculated as follows.

(4) $$NV=\sum_{i=1}^{N}C_{i}$$

**Figure 12:**
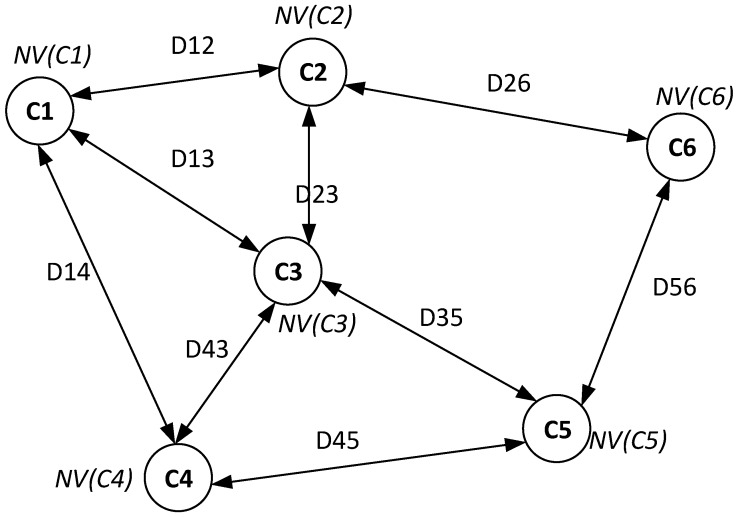
Illustration of a network model with six cluster nodes.

We denote $D_{ij}$ as the distance between node $C_{i}$ and node $C_{j}$. We then model the system as a network topology, as illustrated in Figure 12.

For the path recommender, each cluster node $C_{i}$ maintains a neighbor table containing information of neighbor nodes. Each neighbor table record of node $C_{i}$ for a neighbor node $C_{j}$ consists of the following properties:

- Neighbor ID.
- Distance $D_{ij}$ from node $C_{i}$ to node $C_{j}$.
- Number of parking violations $NV(C_{j})$ at node $C_{j}$.

The distance between two nodes $C_{i}$ and $C_{j}$ is calculated as the distance between two master nodes of the two corresponding parking areas. Each time a node receives a new event with an updating number of parking violations from sensor data, it will broadcast up-to-date information to neighbor nodes.

### 4.5. Cost Function

We now construct a cost function used for the path recommender. We denote $f_{ij}$ as the cost function, which calculates the cost between two nodes $C_{i}$ and node $C_{j}$ where parking violation events happen. The cost is calculated based on the distance $D_{ij}$, and the number of violations $NV(C_{j})$. $f_{ij}$ is defined as follows:

(5) $$f_{ij}=\lambda *D_{ij}/D_{max}+\eta *\left(NV_{max}^{*}-NV\left(C_{j}\right)\right)/NV_{max}^{*}$$

where $D_{max}$ is the maximum distance between two nodes in the network; $NV_{max}^{*}$ is the maximum number of parking violation in a parking area, equal to the maximum number of parking bays in a parking area; $D_{max}$ and $NV_{max}^{*}$ are two global parameters; *λ* is a coefficient depending on the distance; *η* is a coefficient depending on the number of parking violations; λ,η∈[0,1] and λ+η=1.

The cost $f_{ij}$ plays a role as a weight of the link between node $C_{i}$ and node $C_{j}$. After an officer finishes handling violations in a parking area, the system recommends the next parking area with parking violations for the officer to visit so that the moving path of the officer is within the least cost *f*. The cost *f* is inversely proportional to the number of parking violations. This means that the recommender tends to find a path with a high number of parking violations. The purpose is to increase the number of violations an officer can handle, as well as to increase the number of parking tickets issued within one movement. The cost *f* is directly proportional to the distance between two nodes. This means that the recommender tends to find a parking area with parking violations that is nearby the current position of the officer, to minimize the time required to approach violations.

The values of the two coefficients determine the weight of each parameter (*i.e*., the number of parking violations at the next parking area or the distance to the next parking are). City authorities can determine which parameter is more important based on their goal and then adjust the values of *λ* and *η* accordingly to achieve better expected results. For example, with λ=0, only the number of parking violations is taken into account. In this case, the system tends to select the next parking area with the highest number of parking violations. With η=0, only distance is considered. The system tends to select the nearest parking area with parking violations. By balancing the values of *λ* and *η*, the system tends to select a path to increase the productivity of an officer in finding and handling violations.

### 4.6. Least Cost Path Recommendation Algorithm

The path recommender uses neighbor tables with calculated cost information (*i.e*., using Equation (5)) to determine the next node and recommend the least cost path to which to route the officer. The algorithm for the path recommender is presented as follows.

Given a network topology as a weighted graph Gwith a set of C nodes and a set of weighted links *L*. The weight is determined by the function *f*. An officer O is currently located at node $C_{i}$ and sends a request to find the next parking area to visit. The detail of the algorithm is presented in Algorithm 1.

**Algorithm 1** Least cost path recommendation algorithm.**INPUT:**
G={C,L}, $C_{i}$**OUTPUT:** The next node $C_{next}$ to visit**Initialize:** O –> current location = $C_{i}$, O–>request = 1, min←∞, next hop $C_{next}=\emptyset$**Repeat** **for all**
$C_{j}\in$ neighbor tables of $C_{i}$
**do**  Calculate: $f_{ij}$  **if**
$f_{ij}<min$
**then**   $min=f_{ij}$   update: $C_{next}=C_{j}$  **end**
**if** **end**
**for** **return**
$C_{next}$

After the next node is determined based on the algorithm above, we use the Google Map API to find the shortest path between two parking areas corresponding to the two nodes. The shortest path may depend to the travel mode of the officer. In the experiments, we currently use the walking mode by declaring Google Map API *travelMode: google.maps.TravelMode.WALKING*.

### 4.7. Updating

When a node detects an event and the number of parking violations in the system is changed, the system then updates the status of related nodes. For example, when a violating car is processed by an officer or when a sensor detects a violating car has left without receiving a ticket, the number of parking violations in the corresponding parking area is reduced by one.

### 4.8. Fault-Tolerant Mechanism

The following mechanism is proposed to assist officers when they are not in any parking space. For example, when an officer is moving towards a selected destination node, a new event is reported that violating cars at the destination node have left and there is currently no violating cars in the destination node. In such a case, the system should make another recommendation for the officer, and the officer should re-select another parking area to inspect. In particular, the system triggers a fault-tolerant mechanism as follows. A temporary node is created at the current location of the officer. This node sends a broadcast to other neighbor nodes around to request the neighbor information. Based on the responses from neighbor nodes, the node quickly builds a neighbor table. After that, the same least cost path recommendation algorithm is used to find another next hop.

## 5. Numerical Results

To evaluate the proposed system, we carry out simulations based on the real parking sensor dataset of Melbourne as follows.

### 5.1. System Implementation

We first import all location information of the sensors, parking areas and parking rules of each area from the dataset into a SQL database. The system is then implemented in C#, and simulations are run on a Windows core i5 desktop PC with a process frequency of 3.33 GHz and 8 GB RAM. We use the Google Maps API to calculate the distance between nodes and to request the shortest path between two nodes based on the walking mode.

### 5.2. Distance

For the cost function, we use the Google Maps API to calculate the distance between two nodes. The distance between two nodes depends on the mode of traveling. In our simulations, we use the walking mode (*i.e*., *travelMode: google.maps.TravelMode.WALKING*) and assume officers walk with an average speed of 6 km/h. As a result, the distance between two nodes is the walking distance. The distance between two nodes is obtained by using the Google Map API Distance Matrix Request, which consists of the following parameters: origin (*i.e*., address of node $C_{1}$), destination (*i.e*., address of node $C_{2}$), Google API key and mode (*i.e*., *travelMode: google.maps.TravelMode.WALKING*).

### 5.3. Shortest Path

After the path recommender determines the best next destination to visit, the system recommends a path to the officer. We use the shortest path calculated using the Google Maps API Directions Request, which consists of the same parameters as described above for calculating the distance. Similarly, the walking mode is used.

### 5.4. System Parameters

The detailed parameters used in our simulation are shown in Table 1.

We run simulations based on the real parking sensor data of eight political regions of the city, including four regions with a high volume of parking violations (*i.e*., City Square, Hyatt, Princess Theatre and Chinatown, as shown in Figure 5) and four regions with a low volume of parking violations (*i.e*., RACV, County, Magistrates and Regency, as shown in Figure 5). The purpose is to study the performance of the system in areas with different violation distributions. We use the real number of officers responsible for each region in our simulations. Normally, each political region is equal to one parking region for parking management purposes. Several large political regions, like City Square, Hyatt, Princess and Chinatown, are equal to two parking regions. Therefore, in our simulations, such a large political region is also divided into two parking regions following the real situation. Each parking officer supervises one parking region.

Based on the distance computation, the maximum distance between two nodes $D_{max}$ is 862 m, and the highest number parking bays in a parking area (*i.e*., $NV_{max}$) is 16. Simulations are configured with a balance mode of the two coefficients *λ* and *η*. We assume officers work from 8 a.m. to 8 p.m. In reality, officers may be divided into different working shifts with such working hours. However, in our simulations, all officers are the same, so we use only one officer as a representative user during such working hours. We assume after arriving at the location of a violating car, the time required for an officer to issue a fine ticket is 2 min. After that, he or she will move to another parking violating car in the same parking area or send a path recommendation request to move to another parking area. In real working scenarios, an officer may not focus on the task all of the time, so we assume an officer can have a leisure time of 5 min every working hour. Those numbers are obtained from advice by experienced officers. We configure the system with those realistic numbers to make simulation scenarios similar to the real working environment of officers. We run each simulation with weekly data (*i.e*., data of seven days in a week). For reliability, simulations are repeated for a different ten weeks in a year. The results presented below are the average of ten runs.

### 5.5. Performance Metrics

We define the following performance metrics to evaluate the performance of the proposed system.

- **The average parking violation period** is measured from the time a car starts violating until the time the car is penalized.
- **The average traveling cost (walking distance/fine ticket)** indicates average distance an officer has to travel to issue a fine ticket.
- **The average ticket issued ratio per movement** is the ratio between the number of movements with at least one fine ticket issued and the total number of movements. When an officer moves from a cluster node to another cluster node, we say that the officer makes one movement.
- **Productivity (number of issued tickets per hour)** is the average number of tickets an officer issues within one hour.

### 5.6. Results

We use the above metrics to compare the performance of our proposed system with (1) statistical numbers, which are real numbers obtained in real situations about parking violation management of the city, (2) a traditional random inspection approach in which an officer selects a random parking area to inspect and (3) the random selection of the next node to inspect with violation awareness. Note that the traditional random inspection is the current approach used by officers. However, there is a small difference between the results obtained by our simulation for the traditional random inspection approach and statistical numbers, because in real cases, officers may have experience in finding parking violations, in addition to their random approach. In the third scheme, we assume officers know which parking areas have parking violations (*i.e*., provided by our system), but there is detailed information and no recommendation for the best path from the system; officers then randomly select a parking area with parking violations to inspect. We first compare the results of the four approaches in terms of the average ticket issuing productivity of officers and the average violation period of violating cars. For other results, we compare the proposed system with the two random methods only because statistical results for real cases are not available.

**Figure 13:**
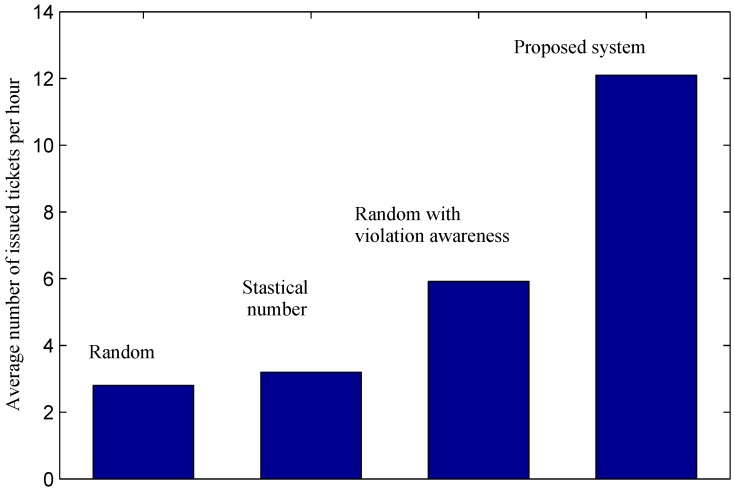
Average number of issued parking tickets per hour by a parking officer.

Figure 13 presents a comparison of the average ticket issuing productivity of officers among different approaches. The random approach shows the lowest productivity with only 2.8 tickets per hour. In this approach, an officer inspects parking areas randomly without any *a priori* information to find parking violations. As a result, the probability that the officer finds a violation is relatively low. Although current officers also inspect parking areas randomly, the productivity in the real case shown by the statistical number is better than the random approach. This may be due to the fact that in real cases, officers have experience at finding parking violations. By providing officers with parking violation information *a priori*, the random method with violation awareness achieves a much better result compared to the traditional random one. In particular, by using this method, the productivity of an officer is doubled with 5.92 tickets per hour compared to the traditional one. The proposed system achieves a considerable improvement in the productivity of an officer. Results show that the proposed system helps increase the number of issued tickets per hour by an officer from 3.2 tickets in the real case to 12.1 tickets, approximately four times. Compared to the random selection with violation awareness, our system achieves over 104% productivity improvement.

**Figure 14:**
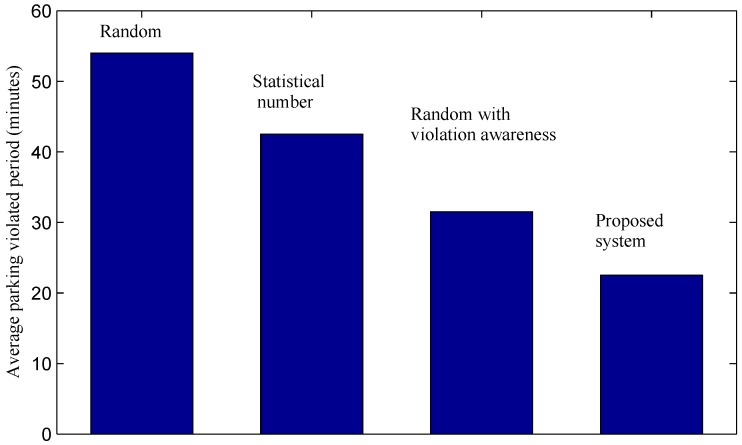
Average parking violation period of violating cars.

Figure 14 shows the average violation period of violating cars until they receive parking tickets. With a low ticket issuing productivity, the random method and the real case witness long parking violation periods. Many cars break parking rules for a long period of over 50 min before they receive tickets. The result indicates that many other cars may have to wait for a long period to find an available on-street parking bay. This leads to other serious issues, such as traffic congestion, the unhappy feeling of visitor or wasting time and money. The results reflect such real issues of current on-street parking in the city. The graph of the third method shows that by providing violation information, officers can find violations faster. As a result, the average parking violation period is reduced to 31.5 min in the random method with violation awareness. The proposed system achieves the shortest average violation period with only 22.5 min. This significant reduction in violation periods is meaningful in an attempt to reduce the issues as discussed above. By using the proposed system, parking violating cars suffer from a high probability of being penalized within a short period after violating. Such an achievement may increase the awareness of drivers about the importance of parking rules, thus potentially helping to reduce the number of parking violations.

**Figure 15:**
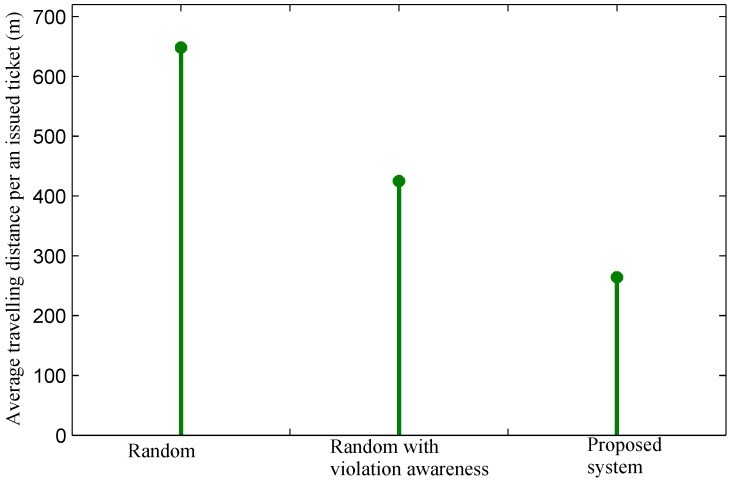
Average travelling distance of officers per an issued ticket in regions with high violation volumes.

We are now interested in exploring the average traveling cost of officers in finding a violation and issuing a parking ticket. The average traveling cost of officers highly depends on the parking violation distribution of regions, so we differentiate results in regions with a high violation volume and regions with a low violation volume, as specified above. In regions with a high violation volume, the average traveling distance of officers using the random method to find a violation and to issue a ticket is over 640 m, while that in the cases of the random method with violation awareness and the proposed system are 425 m and 264 m, respectively, as shown in Figure 15. The results point out that with violation information obtained from sensor data, the traveling cost of officers is reduced greatly. The proposed system achieves a significantly better result than the random approach with violation awareness because the proposed system tends to recommend the best next hop with the lowest cost and a high number of violations to officers. As a result, our system enables officers to find nearby parking areas with possibly multiple parking violations. Within the movement from the current location to next parking area, officers may find multiple violations and issue more than one parking ticket.

**Figure 16:**
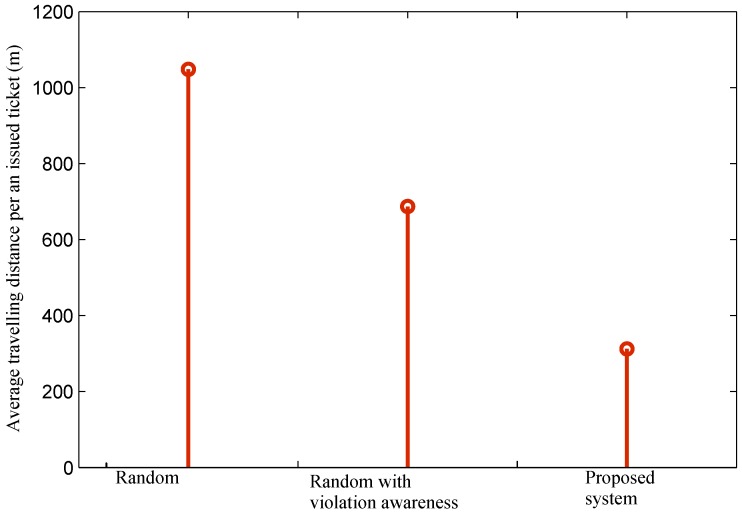
Average travelling distance of officers per an issued ticket in regions with low violation volumes.

Results for regions with a low number of parking violations are shown in Figure 16. The average traveling distance per an issued ticket in regions with a low parking violation volume is considerably higher than that with a high volume. The result of the random method in this case is almost doubled compared to that with a high volume. The reason is that the probability to find violations per movement is lower. As a result, officers may have to visit multiple parking areas before they find a parking violation and issue a ticket. In the case of the random method with violation awareness and the proposed system, although parking areas with violations are known *a priori*, their location may be farther, and the probability of a violating car leaving before officers arrive is also higher.

**Figure 17:**
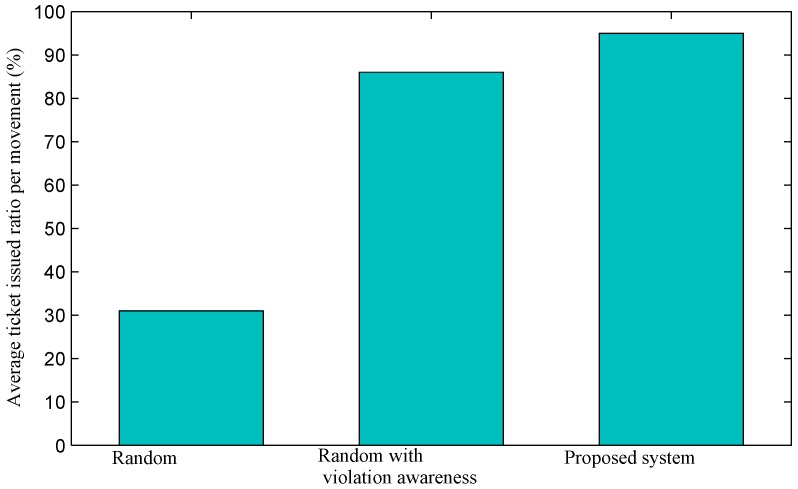
Average ticket issued ratio per a movement (*i.e*., from a cluster node to another cluster node) of officers in regions with high violation volumes.

**Figure 18:**
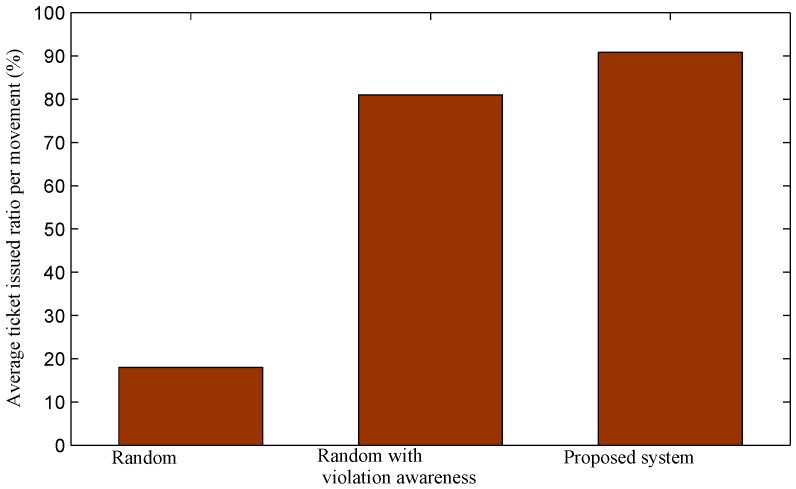
Average ticket issued ratio per a movement (*i.e*., from a cluster node to another cluster node) of officers in regions with low violation volumes.

The results in Figure 15 and Figure 16 can also be explained by using the results that are presented in Figure 17 and Figure 18. Figure 17 illustrates the average ticket issued ratio per a movement of officers in regions with a high violation volume. In other words, the ratio is the probability that an officer makes a movement from the current node to another node (*i.e*., another parking area) and of finding at least one parking violation. The figure reveals that the random approach is inefficient because the ratio is relatively low at approximately 30%. This does mean that an officer may have to inspect several parking areas before he or she luckily finds a parking violation and issues a ticket. The ratios in the cases of the random method with violation awareness and the proposed system are much higher: over 86% with the former and over 95% with the latter. The proposed system selects the next parking area that has the least cost and a high number of violations, so that the ratio is high, and the probability that violating cars will leave before officers arrive is low.

The average ticket issued ratio per a movement of the traditional random method in regions with a low violation volume is really small, below 20%, as shown in Figure 18. As a result, the average traveling cost per an issued ticket of officers is high, as shown in Figure 16. Results for the other two approaches are only slightly reduced. The proposed system achieves the highest ratio and always maintains the ratio over 90%.

The results above demonstrate that the proposed system is helpful in assisting officers in finding on-street parking violations and issuing tickets efficiently. In particular, the system not only improves the productivity of officers considerably, but also helps reduce the traveling cost of officers and reduce the average violation period of violating cars.

### 5.7. Analyzing the Impact of λ and η

**Figure 19:**
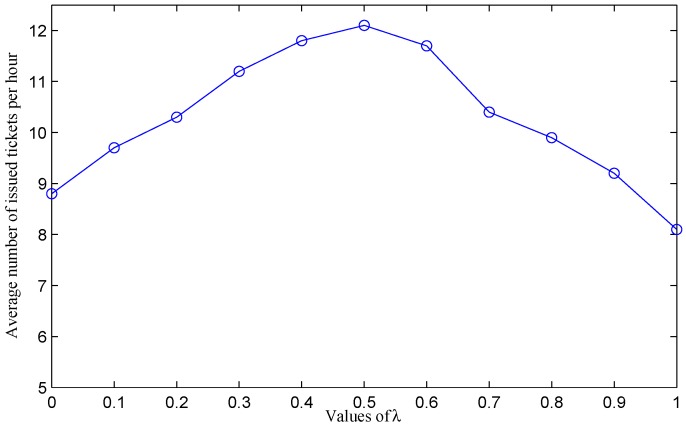
Average ticket issuing productivity of officers under different values of *λ* and *η* (*λ* + *η* = 1).

We are now interested in analyzing the impact of *λ* and *η* on the average ticket issuing productivity of officers. We repeat the above simulations for different values of the two coefficients *λ* and *η* (*λ* + *η* = 1). Figure 19 shows the average number of issued tickets per hour per officer under different values of *λ*. When λ=0 and η=1, the average ticket issuing productivity of officers is 8.8 ticket/hour. In this case, the system selects the next hop based on the number of parking violations only. When we increase the value of *λ*, the average productivity of officers also increases. With λ>0, the system takes traveling cost (*i.e*., distance) into account to select the next hop. As the objective of this paper is to improve the productivity of officers, both the number of violations and the traveling cost should be considered together. Figure 19 indicates that the system achieves better results when both *λ* and η>0. Officers achieve the highest productivity at λ=0.5 and η=0.5 when the two parameters are considered equally. After achieving the peak value, the productivity values fall down when we increase the value of *λ*.

Depending on targets of the system, we can determine which parameter (*i.e*., the number of violations or the distance) is more important and adjust the values of *λ* and *η* accordingly. With λ=0 and η=1, the system witnesses the highest average ticket issued ratio per movement; however, the average traveling distance per movement is also the longest. On the contrary, with λ=1 and η=0, the system achieves the shortest distance per movement of officers; however, the average ticket issued ratio per movement is low.

## 6. Discussion and Conclusions

This work raises a real and crucial issue in parking violation management for smart cities and highlights the necessity of an efficient parking violation management system to assist parking officers. In this work, we first provide actual views about the temporal/spatial distribution of parking violations in a city by carrying out an extensive analysis of a massive parking sensor dataset obtained from real parking sensors. The analysis results show that the parking violation distribution is highly dynamic in the spatial and temporal dimension. As a result, a location-centric cloud-based system is proposed for efficient parking violation management. The system gathers parking sensor data from parking sensors and processes sensor data for parking violation detection on the cloud. Violation information is grouped based on the spatial dimension and distributed to officers based on their location. The system enables officers to easily find parking violations around them. Moreover, a path recommender is designed with the proposed algorithms to find the best path with the least cost and a high number of parking violations for officers. The purpose is to improve the productivity of officers in finding violations and issuing parking tickets. From the theoretical perspective, the system is modeled as a network, and the path finding problem is modeled as the least cost forwarding problem. The problem is then solved by proposing a least cost path recommendation algorithm.

The system is evaluated by simulations on a real sensor dataset and realistic working condition assumptions based on the advice of experienced parking officers. The results show that the system achieves considerable improvements in terms of officers’ productivity, officers’ traveling cost, the probability of finding violation per a movement and the average violation period. Those results indicate that the proposed system potentially enables officers to manage parking violations efficiently, which may help to reduce the number of parking violations and violation periods.

### Limitations and Future Work

Although preliminary results indicate the considerable efficiency and potentiality of the proposed system, experiments are conducted by simulations only. The reason is that the authors have not had permission to access the real parking sensor system for experiments. Parking sensor systems are owned by city governments (*i.e*., Seoul, Melbourne, *etc*.) and are not opened to public. From the results obtained through this study, we plan to collaborate with city governments and to obtain permission to access real parking sensor networks for experiments and implementations of the system. This work is the first step in an attempt to raise the issue and to design an efficient parking violation management system. Similar to the smart parking management problem, we believe that there are other ways to design and improve the efficiency of the parking violation management system. For example, we plan to extend the model to be a multiple hop network and to determine a multiple hop path through multiple parking areas to maximize the number of issued tickets by an officer. We will investigate a tool to support city governments with planning human resources and region division for efficient parking violation management with the minimum number of officers. We also plan to extend the traveling mode of officers to multi-modes (*i.e*., driving, walking, bicycling, *etc*.) and to consider traveling speed based on real traffic conditions. Currently, we use a balancing mode between the two coefficients of *λ* and *η*. To discover different targets of city governments in parking violation management, we will have more discussions with parking officers. Based on that, we will do experiments with different values of the coefficients to achieve their goals.

## Figures and Tables

**Table 1:** Simulation parameters.

Parameter	Value
*λ*	0.5
*η*	0.5
dataset	weekly dataset
number of regions	8
number of officers	12
$D_{max}$	862 m
$NV_{max}$	16
walking speed	6 km per hour
time for issuing a violation ticket	2 min
daily working time period	8 a.m. to 8 p.m.
leisure time of an officer every hour	5 min
